# Enhanced Cytotoxicity of [10]-Gingerol-Coumarin-Triazole Hybrid as a Theranostic Agent for Triple Negative Breast Cancer

**DOI:** 10.1021/acsmedchemlett.4c00596

**Published:** 2025-02-10

**Authors:** Arthur Deponte Zutião, Bianca Cruz Pachane, Paulo Sérgio Gonçalves Nunes, Herika Danielle Almeida Vidal, Heloisa Sobreiro Selistre-de-Araujo, Arlene Gonçalves Corrêa, Marcia Regina Cominetti, Angelina Maria Fuzer

**Affiliations:** † Department of Gerontology, Universidade Federal de São Carlos - UFSCar, São Carlos, São Paulo 13565-905, Brazil; ‡ Biochemistry and Molecular Biology Laboratory, Department of Physiological Sciences, Universidade Federal de São Carlos - UFSCar, São Carlos, São Paulo 13565-905, Brazil; § Molecular Oncology Research Department, Barretos Cancer Hospital, Barretos, São Paulo 14784-400, Brazil; ∥ Department of Chemistry, Universidade Federal de São Carlos - UFSCar, São Carlos, São Paulo 13565-905, Brazil

**Keywords:** triple-negative breast cancer, [10]-gingerol, coumarin, theranostic

## Abstract

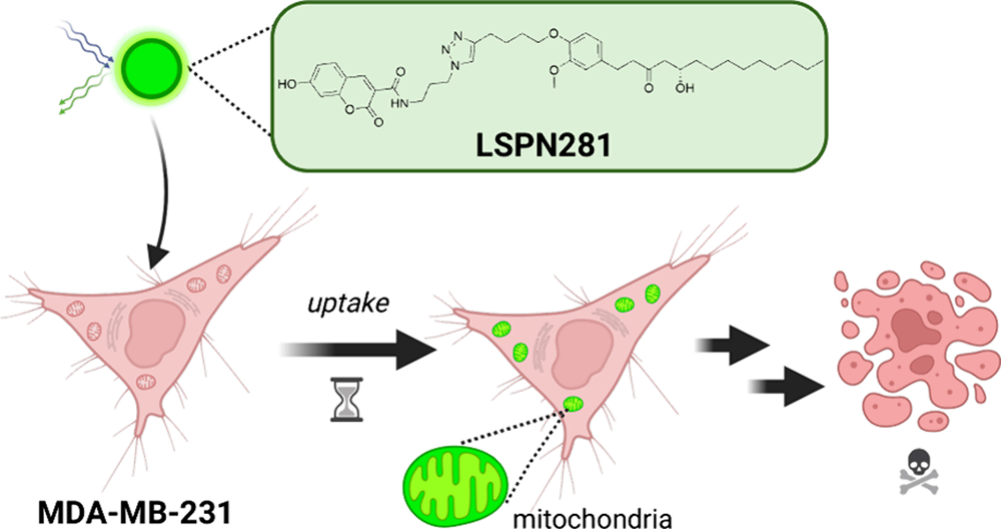

A leading cause of death worldwide, breast cancer is the second most prevalent cancer in women. Triple-negative breast cancer is an aggressive subtype that lacks targeted therapies and requires novel therapeutic approaches in clinical practice to improve the overall survival. Theranostic agents that integrate diagnostic and therapeutic capabilities in a single entity are promising strategies for personalized cancer management. Hybrid compounds combining biologically relevant moieties with different modes of action can enhance cytotoxicity and improve pharmacological properties. We focus on a hybrid containing coumarin, triazole, and [10]-gingerol, a compound with known antimetastatic potential in TNBC. The LSPN281 hybrid exhibited superior cytotoxic activity in a TNBC cell line *in vitro* compared to the individual coumarin and [10]-gingerol controls. Additionally, the hybrid shows enhanced cellular uptake and mitochondrial localization, suggesting its potential as a theranostic agent for TNBC.

Cancer is one of the leading causes of death worldwide, accounting for 20 million new cases in 2022 and 9.7 million deaths globally.^1^ Predictions suggest that over 35 million new cancer cases will be diagnosed by 2050 due to population growth, aging, and increased exposure to pro-tumoral factors.^1,2^ In women, breast cancer is the second most prevalent neoplasia, affecting 2.3 million patients and comprising 11.6% of female tumors.^2^ Breast cancers are categorized based on their expression of estrogen and progesterone receptors (ER, PR), human epidermal growth factor receptor 2 (HER2), and the Ki67 proliferation index.^3,4^ The lack of ER, PR, and HER2 expression occurs in 15% of breast tumors and characterizes the triple-negative breast cancer (TNBC) subtype, known for its aggressiveness and insensitiveness to targeted therapy.^5,6^ TNBC treatment relies on cytotoxic agents that, while effective, are also prone to relapse in 40% of cases.^5^

In this scenario, novel TNBC therapeutic approaches are required to improve the overall patient survival. One current strategy is the employment of hybrid compounds with enhanced cytotoxic activities, where biologically relevant moieties with different modes of action are combined in drugs with improved pharmacological properties. These hybrid drugs, which target multiple pathways involved in cancer cell growth, have shown promising results in cancer therapy as theranostic agents.^7,8^ The emergence of compounds that combine diagnostic and therapeutic properties in a single entity offers a novel approach to personalized medicine, integrating cancer detection, monitoring, and treatment.^9−11^ Simultaneously, theranostic agents also show the ability to selectively target tumors, deliver therapeutics, and provide real-time feedback on the treatment response.^12^ For instance, the integration of imaging and therapeutic modalities in theranostic agents can improve diagnostic accuracy, guide therapy selection, and enhance treatment efficacy, ultimately leading to better patient outcomes.^9,10,12,13^

Coumarin-1,2,3-triazole-based hybrid molecules have shown great anticancer potential and, coupled with their fluorescence properties, are an important tool for *in vitro* investigations.^14−18^ The pharmacokinetics of 10G was also investigated in humans, and the administration of high doses was considered safe.^20^ We have previously described the antimetastatic potential of [10]-gingerol (10G) in TNBC, suggesting it is a safe compound for complementary therapy in metastatic breast cancer.^19^ In this study, we focused on investigating the internalization of 10G-coumarin-triazole hybrid LSPN281 in a TNBC cellular model *in vitro*.

Following the process described in Scheme 1, coumarin **6** was obtained from 2,4-dihydroxy benzaldehyde (**4**) and meldrum acid (**5**)^21^ and reacted with 3-azidopropan-1-amine using 2-(1H-benzotriazole-1-yl)-1,1,3,3-tetramethylaminium tetrafluoroborate (TBTU) and triethylamine furnishing the azide **7** in 76% overall yield. In parallel, 10G (**8b**) reacted with 6-iodohex-1-yn and Cs_2_CO_3_ to form compound **9b**. Finally, the click reaction of azide **7** with alkyne **9b** furnished the desired compound LSPN281 (**10b**). A second compound, LSPN280 (**10a**), was synthesized to contain chlorine as the aryl substituent instead of the natural product 10G for evaluating the influence of coumarin and triazole rings on 10G antitumor activity. We have also evaluated methyl 10G (**11**) for the potential decrease in antitumoral activity caused by alkylation of the phenolic group as already shown for [6]-gingerol (6G).^22^ All compounds were >95% pure by HPLC, and the nuclear magnetic resonance (NRM) spectra for each compound are available in the Supporting Information.

**Scheme 1:**
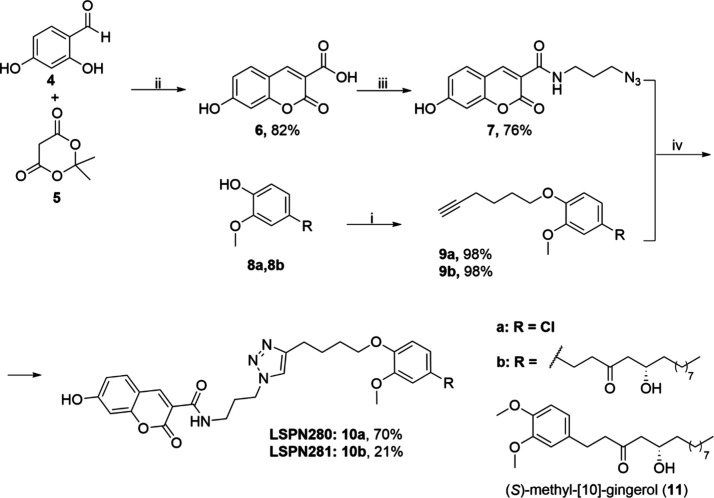
Synthesis of Hybrids LSPN280 and LSPN281 Reagents: (i) K_2_CO_3_, H_2_O; (ii) TBTU, triethylamine, 3-azidopropan-1-amine, DMF; (iii) 6-iodohex-1-yn, Cs_2_CO_3,_ THF; (iv) sodium ascorbate, CuSO_4_, DMF.

***Caution!** 3-azidopropan-1-amine is a flammable liquid (category 3) with acute toxicity (category 3, oral). N,N,*N*′,*N*′-Tetramethyl-O-(benzotriazol-1-yl)uronium tetrafluoroborate (TBTU) is a flammable solid (category 1) with skin and eye irritation (category 2). N,N-dimethylformamide (DMF) is a flammable liquid (category 3) with acute toxicity (category 4).*

The impact of met10G conjugation on the natural fluorescence of LSPN280 was investigated by absorbance at a concentration of 5 μM, diluted in PBS. The novel molecule, hereby named LSPN281, showed a considerably higher mass and broader absorbance spectrum compared to those of the LSPN280 precursor (Figure 1A). The spectral behavior of both compounds is similar, peaking at an optical density of 360 nm. From the maximum peak, the optimal excitation wavelength was determined to trace the fluorescence spectrum of each compound. Under the same fluorescence peak at 460 nm, the emission of hybrid LSPN281 is considerably higher than that of LSPN280 (Figure 1B), suggesting that the synthesis of LSPN281 enhances the optical properties of its precursor, facilitating the investigation of intracellular entry and distribution *in vitro.*

**Figure 1:**
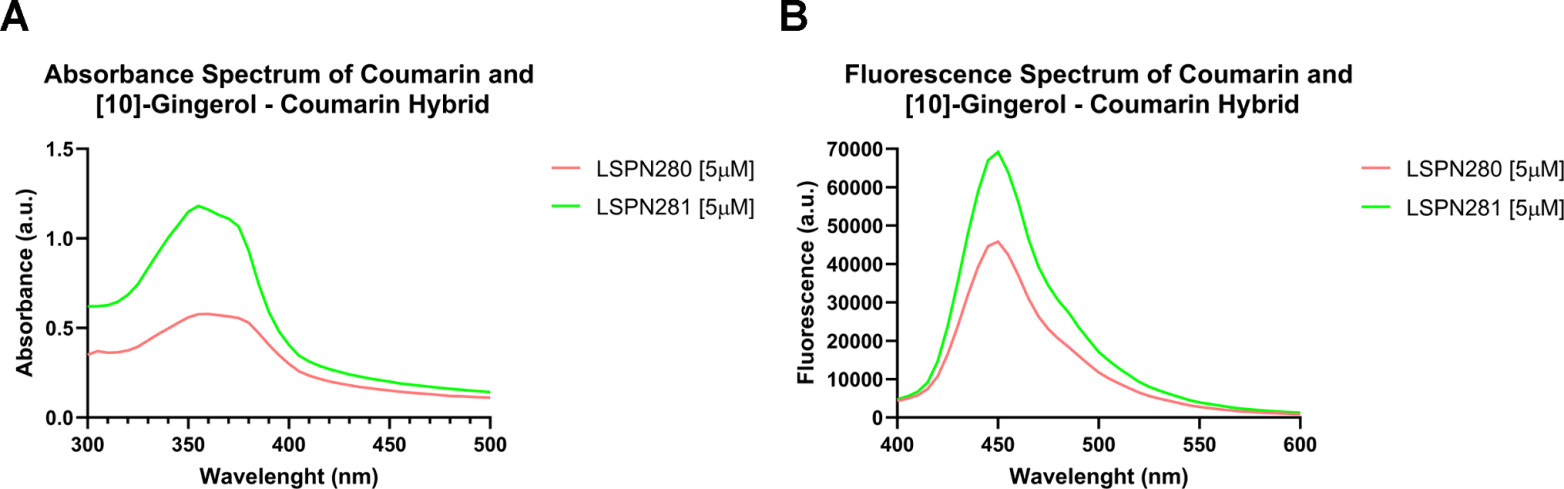
(A) Absorbance and (B) fluorescence spectra of LSPN280 and the LSPN281 hybrid, derived from the combination of met10G and LSPN280.

The half-maximum inhibitory concentrations (IC_50_) of met10G, LSPN280, and LSPN281 were determined using a resazurin-based cytotoxicity assay, leading to the values shown in Table 1. The LSPN281 hybrid exerts an enhanced effect on the natural cytotoxicity of met10G, reducing its IC_50_ by half within 24 h and up to 10-fold after 48 h. The enhancement of the cytotoxic effect of met10G supports the proposal of LSPN281 as a theranostic agent, with increased fluorescence and cytotoxicity compared to LSPN280 and met10G.

**Table 1:** Optimization of met10G, LSPN280, and LSPN281 Treatment in TNBC Cell Line MDA-MB-231

cell line: MDA-MB-231	IC_50_ (μM) ± SE	
treatments	24 h	48 h
LSPN280	115.8 ± 18.3	103.4 ± 7.8
Met10G	45.6 ± 2.9	26.9 ± 1.4
LSPN281	19.1 ± 2.5	2.3 ± 0.1

The natural fluorescence of the hybrids enabled our investigation of their internalization in TNBC cell line MDA-MB-231. Using an epifluorescent microscope containing a FITC filter with a 488 nm excitation laser, the internalization of LSPN280, LSPN281, or met10G was evaluated for up to 2 h. It is important to state that cells exhibited an expected low level of autofluorescence, as detected in the untreated group, and the mean values were used for threshold images before quantification. After 30 min of treatment, the compounds were located under in the cellular perimeter and detected intracellularly throughout the assay duration (Figure 2A). The quantification of fluorescence observed in cells increased signals after 30 min of treatment with met10G, LSPN280, and LSPN281, compared with the untreated control (Figure 2B). The treatment with LSPN281 was 3-fold more effective than met10G, considering the intensity and area of fluorescence. After 1 h of treatment, met10G and LSPN280 levels were reduced to baseline values, whereas LSPN281 fluorescence persisted (Figure 2C).

**Figure 2:**
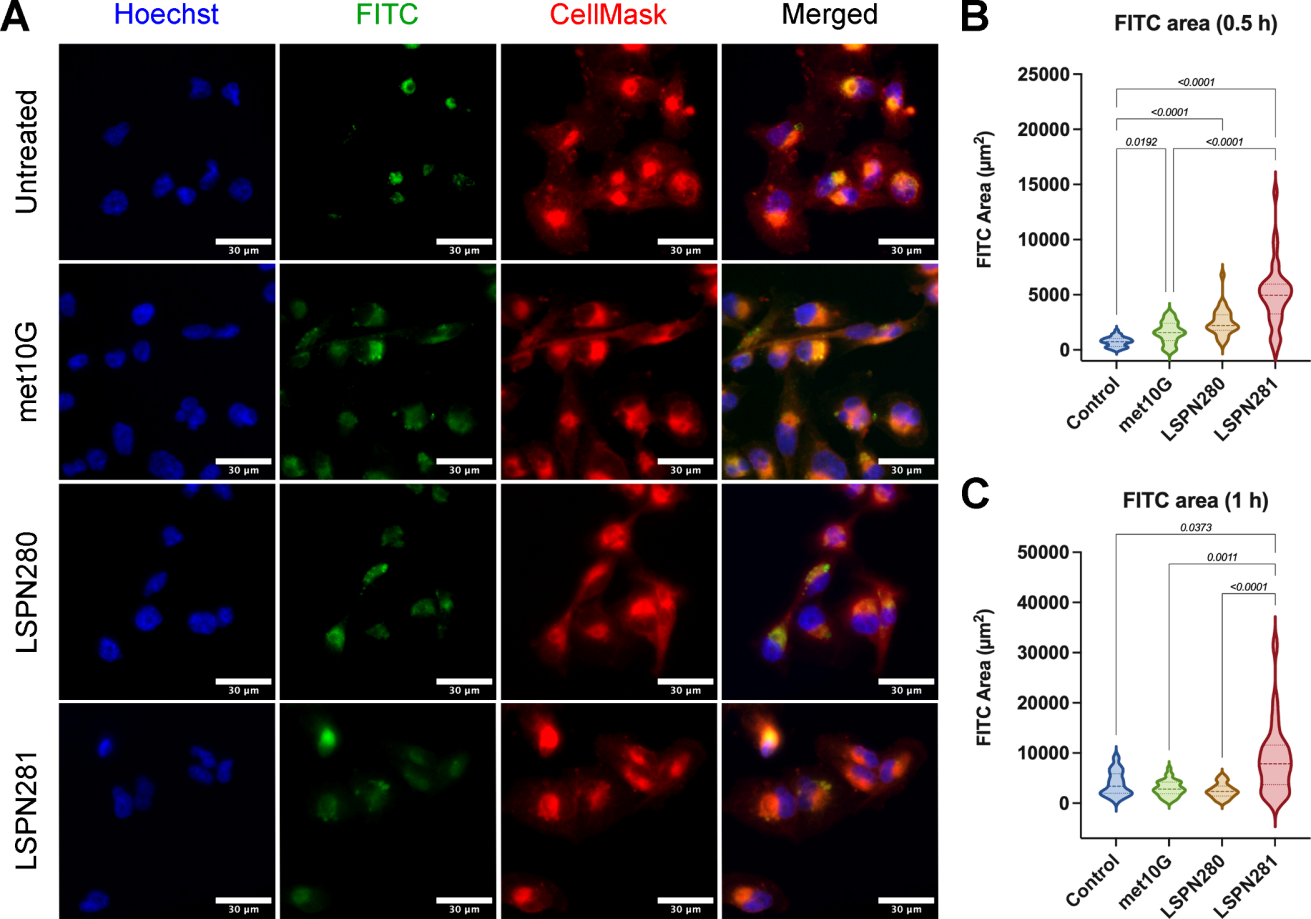
Hybrid compound association in TNBC cells. (A) Representative images of MDA-MB-231 cells stained with Hoechst (nuclei, blue) and CellMask (membrane, red) with compounds (met10G, LSPN280, or LSPN281) detected in the FITC filter (green) following a 30 min treatment. Scale bar: 30 μm. (B,C) Quantification of the green fluorescent area detected in cells per group, in μm^2^, after (B) 30 min and (C) 1 h of interaction with the compound. Statistically significant *p* values are displayed on top of the comparisons.

To determine whether the hybrids are taken up by mitochondria, we proceeded with a confocal imaging approach of the same uptake experiment. The fluorescence from the FITC and TxRed channels was gated to avoid channel leak-through, and a baseline threshold was determined based on the untreated control, generating orthogonal reconstitutions for analysis (Figure 3A).

**Figure 3:**
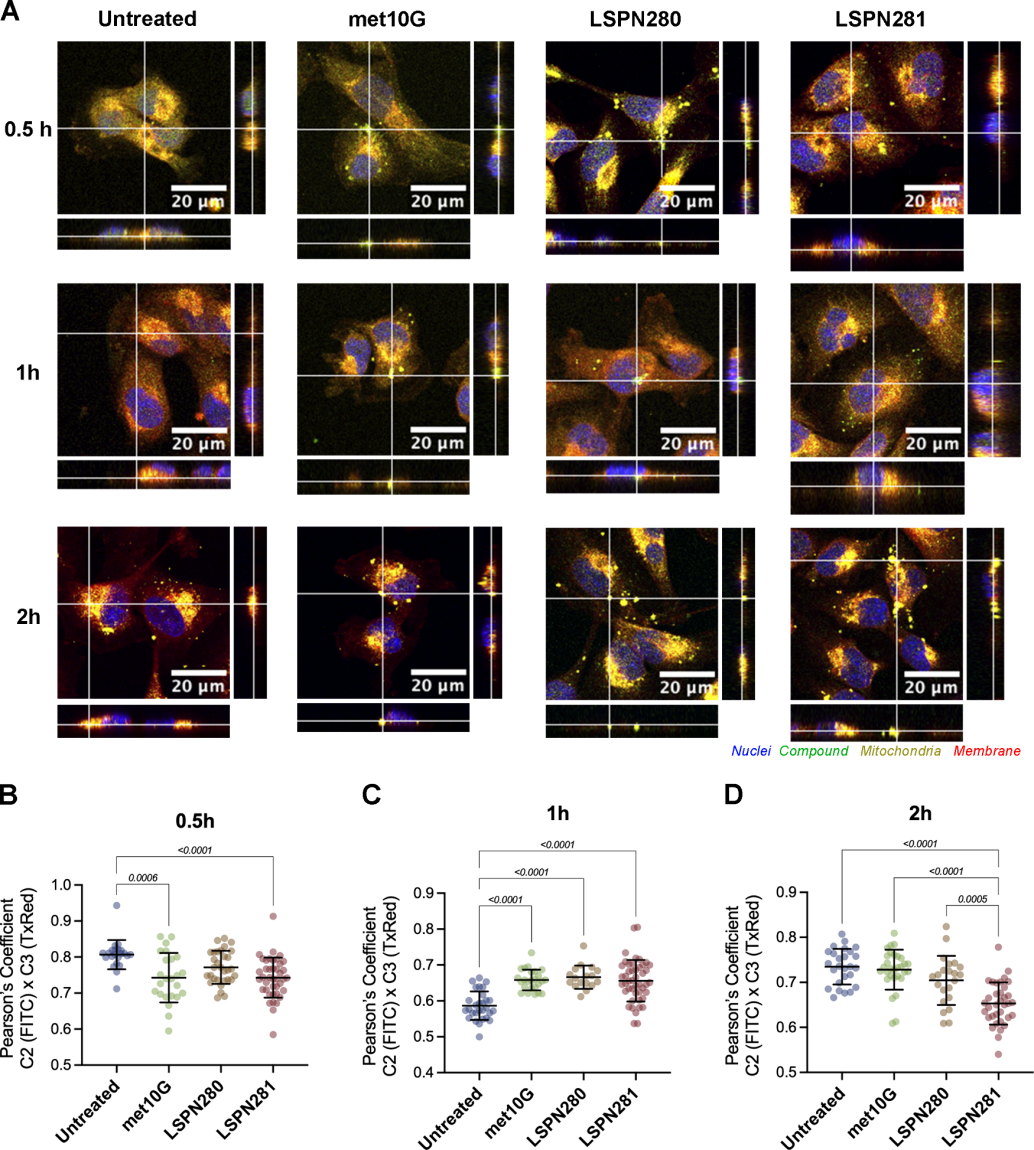
met10G, LSPN280, and LSPN281 colocalization with mitochondria. (A) Orthogonal views of MDA-MB-231 cells stained with Hoechst (nuclei, blue), MitroTracker (mitochondria, yellow), and CellMask (membrane, red) with treatments (met10G, LSPN280, or LSPN281) detected in the FITC filter (green). Scale bar: 20 μm. (B–D) Pearson’s colocalization coefficient between channels 2 (FITC) and 3 (TxRed), determined from Z-stacks of groups after (B) 30 min, (C) 1 h, or (D) 2 h of interaction with the compounds. Statistically significant *p* values are displayed in brackets.

After 30 min of treatment with either compound (met10G, LSPN280, or LSPN281), cells exhibited a diffuse layer of green fluorescence, with small clusters inside of cells and little interaction with mitochondria. This was further evidenced by the determination of Pearson’s colocalization coefficient, which was reduced in the met10G and LSPN281 groups in comparison to the untreated control (Figure 3B). One hour following the treatment, the fluorescence profile changed to encompass small blebs in matching locations to mitochondria, and the colocalization coefficient suggested an increase in the overlapping between the two channels (Figure 3C). After 2 h, the green fluorescence appeared more clustered and spread out throughout the cells, and the colocalization indexes were once again reduced, suggesting compound metabolization (Figure 3D).

Previous studies have demonstrated the antitumoral potential of 10G in TNBC by inducing apoptosis, inhibiting several steps in the metastatic cascade, reversing the malignant phenotype of TNBC, and reducing metastasis formation in syngeneic orthotopic mouse models.^19^ In this study, we further demonstrate the potential of a gingerol–coumarin–triazole hybrid as a promising theranostic agent for TNBC. LSPN281 was demonstrated to enhance cytotoxic activity against TNBC, associating with cells and being metabolized more efficiently over time compared to met10G or LSPN280. These results demonstrate that LSPN281 is a powerful tool to comprehend how 10G-derived compounds influence tumoral metabolism.

10G is a natural product from ginger (*Zingiber officinale* Roscoe), whose medicinal applications have been explored by Eastern culture over centuries.^23^ The biologically active constituents of ginger show great potential in cancer therapy, particularly 6G and 10G. While 6G is the primary active substance in ginger, derivative compounds obtained by substituting the meta position of the triazole ring showed better cytotoxic activity than the original molecule.^8^ Fewer studies have investigated 10G as an antitumoral and antimetastatic molecule but with promising results. Its cytotoxicity was selective toward TNBC cell line MDA-MB-231, compared to the nontumor MCF-10A cell line, with an IC_50_ determined at 12.1 ± 0.3 μM.^24^ Our study showed that the gingerol–coumarin–triazole hybrid LSPN281 had an approximately 5-fold reduction in IC_50_, compared with met10G, suggesting that it is a more efficient molecule for TNBC cytotoxicity.

The biological activity of 10G and its hybrids is very much unknown, but ginger constituents show a tight association with essential cell functions. Previous reports have suggested that epigenetic regulation, chromatin modulation, cell migration, and morphology have been altered by gingerol compounds.^25^ 10G has induced apoptosis in TNBC *in vitro* by increasing caspase 9 activation and reducing EGFR and integrin β1 expression, hinting at the activation of an intrinsic apoptosis pathway.^19,26^ Most apoptotic stimuli occur through the mitochondrial pathway, where the mitochondrial outer membrane permeabilization (MOMP) activates caspase 9 upon cytochrome *c* signaling and apoptosome formation.^27^ Hence, targeting mitochondrial function has emerged as a promising approach in cancer therapy.^28−30^ Our confocal microscopy analysis revealed that the LSPN281 was taken up by TNBC cells and colocalized with mitochondria after 1 h, suggesting a potential mechanism of action involving mitochondrial targeting. Nevertheless, as we continue to explore the therapeutic and diagnostic potential of this novel gingerol–coumarin–triazole hybrid, further studies are needed to elucidate its precise mechanism of action and evaluate its efficacy.

Furthermore, the persistent fluorescence of LSPN281 within TNBC cells over time highlights its potential as a theranostic agent, enabling both therapeutic and diagnostic applications. The ability to visualize the compound’s localization and interaction with TNBC cells could be a first step in developing more personalized and targeted treatment strategies,^31−33^ which is one of the significant issues in the clinical approach to TNBC patients.^5^ The current approach to early stage TNBC involves the administration of anthracyclines and taxanes. While no targeted therapy has emerged, candidates targeting the PI3K/AKT/mTOR pathway, EGFR, Notch, and polyADP-ribose polymerase have been described.^5,34^ We believe that LSPN281 has the potential to function as a theranostic agent, enhancing personalized medicine approaches in the management of TNBC.

In conclusion, the results presented in this study show the potential of the gingerol–coumarin–triazole hybrid as a theranostic agent for triple-negative breast cancer. The LSPN281 exhibited enhanced cytotoxic activity and selective association with TNBC cells. The confocal microscopy analysis revealed that the hybrid was taken up by the TNBC cells and colocalized with mitochondria, reinforcing the mechanism of action involving mitochondrial targeting. Furthermore, the persistent fluorescence of LSPN281 within TNBC cells over time indicates its potential as a theranostic agent. These findings show the potential to develop more personalized and targeted treatment strategies for TNBC in the future.

## Experimental Procedures

### Chemistry

All reagents were purchased from Sigma-Aldrich and Merck. Solvents were obtained from commercial sources and treated as recommended by the manufacturers. The complete method of chemical synthesis is described in the Supporting Information.

### Cell Culture and Reagents

Triple-negative breast adenocarcinoma cell line MDA-MB-231 was acquired from the Rio de Janeiro Cell Bank (BCRJ) and cultured in Leibovitz-L15 cell medium (VitroCell) supplemented with 10% fetal bovine serum (FBS, Thermo-Fisher Scientific). Cells were maintained under sterile conditions at 37 °C and subcultured after 80–90% confluency was achieved with trypsin-EDTA (VitroCell). Stock solutions of the hybrid molecule LSPN281, the fluorophore probe LSPN280, and methylated [10]-gingerol (met10G) were prepared with DMSO to a final concentration of 10 mM. Cell treatment never surpassed a final concentration of 0.5% DMSO.

### Determination of LSPN281, LSPN280, and met10G Half-Maximum Inhibitory Concentration (IC_50_)

MDA-MB-231 cells (10^4^ cells/well) were seeded in six technical replicates using 96-well plates in complete medium. The plates were sealed with Parafilm M for incubation at 37 °C and 24 h. After cell adhesion, the medium was replaced with a serial dilution of each compound, starting from 250 to 0.98 μM, halving each time. After a 24 h incubation with the treatment, the medium was replaced to contain 10% resazurin solution (0.1 mL/mL, #199303, Sigma-Aldrich) and maintained at 37 °C for 4 h. In parallel, we maintained a blank, no-cell control and a 100%-reduced control. The supernatant was transferred to a new 96-well black plate with a clear bottom. Media fluorescence was measured by a plate reader (λ_Exc_ 540 nm, λ_Em_ 685 nm; BioTek Synergy H1). Results from three distinct experiments are displayed as percentage of cell death and plotted in a linear regression for determining the half-maximum inhibitory concentration.

### Absorbance and Fluorescence Spectra of LSPN281, LSPN280, and met10G

LSPN281, LSPN280, and met10G were diluted to a final concentration of 10 μM in phosphate-buffered saline (PBS) and plated in triplicate in a black 96-well plate with a clear bottom. Using a plate reader (Biotek Synergy H1), the absorbance was measured from 400 to 800 nm for each compound, alongside PBS as a vehicle control and empty wells as blank controls. The excitation wavelength of each compound was determined as the peak of the plot of absorbance spectra. In a second set of plates, the fluorescence spectra were evaluated starting at 30 nm more extended than the absorption peak to avoid Rayleigh scattering up to 700 nm. Results were combined in plots using GraphPad Prism (version 8).

### Uptake Assay

Using eight-well chamber slides (Thermo-Fisher Scientific), MDA-MB-231 (1.9 × 10^4^ cells/well) was seeded in a supplemented medium for 24 h at 37 °C. After adhesion, cells were either treated with LSPN281, LSPN280, or met10G (5 μM) in technical duplicates for 0.5, 1, 2, and 4 h at 37 °C. An untreated control was kept in parallel. Cells were stained with CellMask Deep Red Plasma Membrane Stain (C10046, Invitrogen), MitoTracker Red FM (M22425, Invitrogen), and Hoechst (H3570, Life Technologies) for 30 min at RT. Cells were washed with fresh PBS for 5 min at RT and fixed with paraformaldehyde (4% PFA) for 20 min at RT. After fixation, the cells were washed twice with cold PBS-glycine for 15 min. The chamber scaffold was detached, and slides were assembled with coverslips using a Fluoromount (Thermo-Fisher Scientific). Slides were left to dry overnight at 4 °C for microscopy analysis.

### Epifluorescence Microscopy

Slides were observed by using a high content screening system under 40× magnification (ImageXpress Micro XLS, Molecular Devices). A total of 32 images were acquired from the center of the wells using filters for DAPI, FITC, TxRed, and Cy5, with a minimum laser exposure of 10 ms. Laser intensities for each channel were kept consistent throughout groups and time points. Scaled images from the FITC channel were quantified in FIJI,^35^ using a threshold range of 863–65 535 to create binary images. Representative images were cropped from the originals, maintaining selection coordinates (Supporting Information 10).

### Confocal Microscopy and Colocalization Analysis

Slides were evaluated in an LSM880 FAST Airyscan confocal microscope (Carl Zeiss) at 40× magnification. Cells were imaged using a pinhole of 1 AU and gated channels for DAPI (410–508 nm, laser 2.0%, gain master 560), FITC (493–571 nm, laser 6.0%, gain master 800), TxRed (548–620 nm, laser 12.0%, gain master 750), and Cy5 (638–759 nm, laser 2.0%, gain master 500). Z stacks were acquired with 0.8 μm intervals ranging from manually set cell apical and basal points of two distinct sites containing a minimum of 20 cells. Colocalization analysis of FITC and TxRed channels was performed using the plugin BiOP JaCoP from FIJI^22^ while keeping consistent thresholds to determine Pearson’s coefficient (Supporting Information 11).

### Statistical Analysis

Data sets were checked for outliers using ROUT’s test and distribution by D’Agostino–Pearson omnibus K2. Parametric data were evaluated using ANOVA one-way with Tukey’s multiple comparison test. Nonparametric data were analyzed using Kruskal–Wallis analysis of variance with Dunn’s multiple comparison test. In the viability assays, a concentration–response curve was calculated upon fitting the response data to a sigmoidal equation using a four-parameter logistic function:
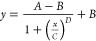
where *y* represents the measured response, *A* is the minimum response, *B* is the maximum response, *C* is the IC_50_, and *D* is the Hill coefficient, which describes the curve’s slope. The IC_50_ value was obtained directly from the fitted concentration–response curve, identified as the point at which the curve reached 50% of the maximum response. Values of *p* < 0.05 were considered statistically relevant. Data analysis and graph design were made on GraphPad Prism (version 10.3.1).

## Supporting Information Available

Spectral characterization methods, data, and additional fluorescence images (PDF)

## Abbreviations

10G: [10]-gingerol
AKT: protein kinase B
DAPI: 4′,6-diamidino-2-phenylindole
EGFR: epidermal growth factor receptor
ER: estrogen receptor
FITC: fluorescein isothiocyanate
HER2: human epidermal growth factor receptor 2
MOMP: mitochondrial outer membrane permeabilization
mTOR: mammalian target of rapamycin
NMR: nuclear magnetic resonance
PI3K: phosphoinositide 3-kinase
PR: progesterone receptor
TNBC: triple-negative breast cancer
