# Supplementary material for: Enhanced Cytotoxicity of [10]-Gingerol-Coumarin-Triazole Hybrid as a Theranostic Agent for Triple Negative Breast Cancer
Source: ACS Med Chem Lett. 2025 Feb 10;16(3):436–43. doi: 10.1021/acsmedchemlett.4c00596 (PMC11912263; doi:10.1021/acsmedchemlett.4c00596)
Supplement: Supplementary file 1 — ml4c00596_si_001.pdf [file ml4c00596_si_001.pdf]

SUPPORTING INFORMATION

Enhanced Cytotoxicity of [10]-Gingerol-Coumarin-Triazole Hybrid as a Theranostic Agent for Triple Negative Breast Cancer

Authors: Arthur Deponte Zutião[1], Bianca Cruz Pachane[2,3], Paulo Sérgio Gonçalves Nunes[4], Herika Danielle Almeida Vidal[4], Heloisa Sobreiro Selistre-de-Araujo[2], Arlene Gonçalves Corrêa[4], Marcia Regina Cominetti[1] and Angelina Maria Fuzer[1*]

Affiliations: [1] Department of Gerontology, Universidade Federal de São Carlos - UFSCar, São Carlos, SP, Brazil; [2] Biochemistry and Molecular Biology Laboratory, Department of Physiological Sciences, Universidade Federal de São Carlos - UFSCar, São Carlos, SP, Brazil; [3] Molecular Oncology Research Department, Barretos Cancer Hospital, Barretos, SP, Brazil; [4] Department of Chemistry, Universidade Federal de São Carlos - UFSCar, São Carlos, SP, Brazil

Corresponding Author: Angelina Maria Fuzer

Address: Universidade Federal de São Carlos – UFSCar, Rodovia Washington Luís, km 235.
CEP 13565-905, São Carlos – SP, Brazil
Email: angelina.fuzer@ufscar.br

Table of contents

1. **Supplementary Information 1. General methods and materials** **S2**
2. **Safety Statement** **S5**
3. **Supplementary Information 2: NMR and MS data for azide (7)** **S5**
4. **Supplementary Information 3: NMR and MS data for compound (9)** **S6**
5. **Supplementary Information 4: NMR and MS data for LSPN280** **S8**
6. **Supplementary Information 5: NMR and MS data for LSPN281** **S9**
7. **Supplementary Information 6: Composition of images from epifluorescence HTS** **S10**
8. **Supplementary Information 7: Composition of confocal imaging and colocalization with mitochondria** **S11**

Supplementary Information 1. General methods and materials

All reagents were purchased from Sigma-Aldrich and Merck. Solvents were obtained from commercial sources and treated as recommended by the manufacturers. Product purification by flash column chromatography used silica gel 60, 230-400 mesh ASTM, silica gel 60 A, 70-230 mesh, and silica gel chromatoplates 60 F254 (Merck). Nuclear Magnetic Resonance (^{1}H and ^{13}C NMR) spectra were recorded on Bruker ARX 400 MHz spectrometers.Chemical shifts (δ) are expressed in ppm referenced by the residual solvent signal and coupling constants (J) in Hertz (Hz). The following abbreviation was used to indicate the multiplicity of signs: s (singlet), bs (broad singlet), d (doublet), t (triplet), q (quadruplet), and m (multiplet).

For 7-hydroxy-2-oxo-2H-chromene-3-carboxylic acid (**6**), 2,4-dihydroxybenzaldehyde (**4**) (589.2 g; 4.27 mmol), Meldrum acid (**5**) (2.5 g; 3.88 mmol), water (90 mL) and K_2CO_3 (107.2 mg; 0.77 mmol) were added to a round flask and stirred for 20 h at RT. The resulting mixture was placed in an ice bath and the precipitate was filtered, washed with water, and dried under vacuum to generate coumarin **6** with 82% yield (657 mg; 3.19 mmol). ^{1}H NMR (400 MHz, DMSO-d_6) δ 12.82 (s; 1H); 11.08 (s; 1H); 8.68 (s; 1H); 7.74 (d; J = 8.5 Hz; 1H); 6.84 (d; J = 8.6 Hz; 1H); 6.73 (s; 1H)[21].

For *N*-(3-azidopropyl)-7-hydroxy-2-oxo-2H-chromene-3-carboxamide (**7**), coumarin **6** (50 mg, 0.24 mmol), TBTU (77.87 mg; 0.24 mmol) and triethylamine (0.034 mL; 0.24 mmol) in DMF (1.5 mL) were mixed for 15 min at 0 ºC. After the addition of 3-azidopropan-1-amine (0.024 mL; 0.24 mmol), the mixture was stirred for 16 h at RT, concentrated under vacuum, and purified by flash column chromatography using Hexane/AcOEt 10-40% as eluent. The desired product **7** was obtained in 76% yield (53 mg; 0.184 mmol). ^{1}H NMR (400 MHz, DMSO-d_6) δ 8.77 (s; 1H); 8.70 (t; J = 5.9 Hz; 1H); 7.81 (d; J = 8.5 Hz; 1H); 6.87 (d; J = 8.6 Hz; 1H); 6.80 (s; 10H); 3.43 – 3.36 (m; 5H); 1.78 (p; J = 6.8 Hz; 2H).

The general procedure for obtaining alkynes **9** involved the mixture of phenol derivative **8**, Cs_2CO_3 (2 equiv.), and THF (1 mL) for 10 min, followed by the addition of 6-iodohex-1-yn (1 equiv.) for 12 h under

stirring at 50 ºC. The mixture was concentrated under vacuum and purified by flash column chromatography using Hexane/AcOEt 9:1 as eluent. The desired compounds (9) were obtained in 95-100% yield.

4-chloro-1-(hex-5-yn-1-yloxy)-2-methoxybenzene (**9a**): 15 mg, 0.06 mmol. ^{1}H NMR (400 MHz, $CDCl_3$) δ 6.84 (s; 3H); 6.78 (d; *J* = 8.4 Hz; 1H); 4.00 (t; *J* = 6.5 Hz; 2H); 3.84 (s; 3H); 2.27 (d; *J* = 6.9 Hz; 2H); 2.01 – 1.89 (m; 3H); 1.72 (d; *J* = 15.0 Hz; 2H). ^{13}C NMR (101 MHz, $CDCl_3$) δ 150.1; 147.3; 125.9; 120.4; 113.9; 112.5; 84.2; 68.8; 56.2; 28.3; 25.1; 18.3.

(*S*)-1-(4-(hex-5-yn-1-yloxy)-3-methoxyphenyl)-5-hydroxytetradecan-3-one (**9b**): 8.5 mg, 0.019 mmol). ^{1}H NMR (400 MHz, $CDCl_3$) δ 6.78 (d; *J* = 7.8 Hz; 1H); 6.71 – 6.66 (m; 2H); 4.00 (t; *J* = 6.4 Hz; 3H; 2.84 (t; *J* = 7.4 Hz; 2H); 2.74 (t; *J* = 7.4 Hz; 2H); 2.61 – 2.44 (m; 2H); 2.27 (td; *J* = 7.2 Hz; *J* = 2.4 Hz; 2H); 1.99 – 1.88 (m; 3H); 1.72 (p; *J* = 6.9 Hz; 2H); 1.52 – 1.20 (m; 17H); 0.87 (t; *J* = 6.6 Hz; 3H). ^{13}C NMR (101 MHz, $CDCl_3$) δ 211.5; 149.6; 147.0; 133.7; 120.2; 113.5; 112.4; 84.3; 77.2; 68.7; 67.8; 56.1; 49.5; 45.5; 36.6; 32.0; 29.7; 29.3; 28.4; 25.6; 25.2; 22.8; 18.3; 14.3.

The general procedure for the click reaction occurred by the mixture of sodium ascorbate (1 equiv.) and DMF to an aqueous solution of $CuSO_4$ (1 mol.L^{-1}) for 2 minutes under stirring at RT. The mixture was transferred to a flask containing the alkyne **9**, and washed with DMF (2 x 0.5 mL), followed by the addition of azide **7** (1 equiv.). The mixture was stirred for 12 h at RT, concentrated under vacuum, and purified by flash column chromatography using Hexane/AcOEt 30-100 % as eluent.

N-(3-(4-(4-(4-chloro-2-methoxyphenoxy)butyl)-1*H*-1,2,3-triazol-1-yl)propyl)-7-hydroxy-2-oxo-2H-chromene-3-carboxamide (**LSPN280**, **10a**): 70 % yield (6 mg; 0.012 mmol). ^{1}H NMR (400 MHz, acetone-d_6) δ 10.00 (s; 1H); 8.77 (s; 2H); 7.84 – 7.68 (m; 2H); 6,.8 – 6.89 (m; 3H); 6.86 – 6.81 (m; 2H); 4.44 (t; *J* = 7.0 Hz; 2H); 4.01 – 3.92 (m; 2H); 3.81 (s; 3H); 3.45 (q; *J* = 7.0 Hz; 2H); 2.76 – 2.69 (m; 2H); 2.18 (p; *J* = 7.0 Hz; 2H); 1.84 – 1.75 (m; 4H).

(*S*)-7-hydroxy-*N*-(3-(4-(4-(4-(5-hydroxy-3-oxotetradecyl)-2-methoxyphenoxy)butyl)-1H-1,2,3-triazol-1-yl)propyl)-2-oxo-2H-chromene-3-carboxamide (**LSPN281**, **10b**): 21% yield (6 mg; 0.008 mmol). ^{1}H NMR

(400 MHz, MeOD) δ 8.73 (s; 1H); 7.82 (s; 1H); 7.65 (d; *J* = 8.7 Hz; 1H); 6.88 (dd; *J* = 8.6 Hz; *J* = 2.3 Hz; 1H); 6.81 – 6.75 (m; 3H); 6.69 (dd; *J* = 8.2 Hz; *J* = 2.0 Hz; 1H); 4.47 (t; *J* = 6.8 Hz; 2H); 4.05 – 3.92 (m; 3H); 3.81 (s; 3H); 3.45 (t; *J* = 6.6 Hz; 2H); 3.36 (s; 1H); 2.83 – 2.71 (m; 6H); 2.61 – 2.46 (m; 2H); 2.31 – 2.15 (m; 2H); 1.90 – 1.75 (m; 4H); 1.42 – 1.38 (m; 3H); 1.29 (s; 13H); 0.92 – 0,88 (m; 3H). ^{13}C NMR (101 MHz, MeOD) δ 211.9; 165.9; 164.7; 163.0; 158.3; 150.9; 149.7; 149.0; 148.1; 135.7; 133.0; 123.5; 121.6; 115.7; 115.0; 114.4; 113.8; 112.7; 103.1; 70.0; 68.9; 56.5; 51.3; 46.2; 38.4; 37.9; 33.1; 31.0; 30.7; 30.5; 30.2; 29.7; 27.0; 26.6; 26.0; 23.7; 14.4.

SAFETY STATEMENT

Caution! 3-azidopropan-1-amine is a flammable liquid (category 3) with acute toxicity (category 3, oral);

Caution! N,N,N',N'-Tetramethyl-O-(benzotriazol-1-yl)uronium tetrafluoroborate (TBTU) is a flammable solid (category 1) with skin and eye irritation (category 2);

Caution! N,N-dimethylformamide (DMF) is a flammable liquid (category 3) with acute toxicity (category 4);

Supplementary Information 2: NMR and MS data for azide (7)

A

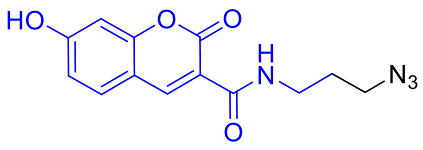

Chemical Formula: $C_{13}H_{12}N_4O_4$
Exact Mass: 288,09

B

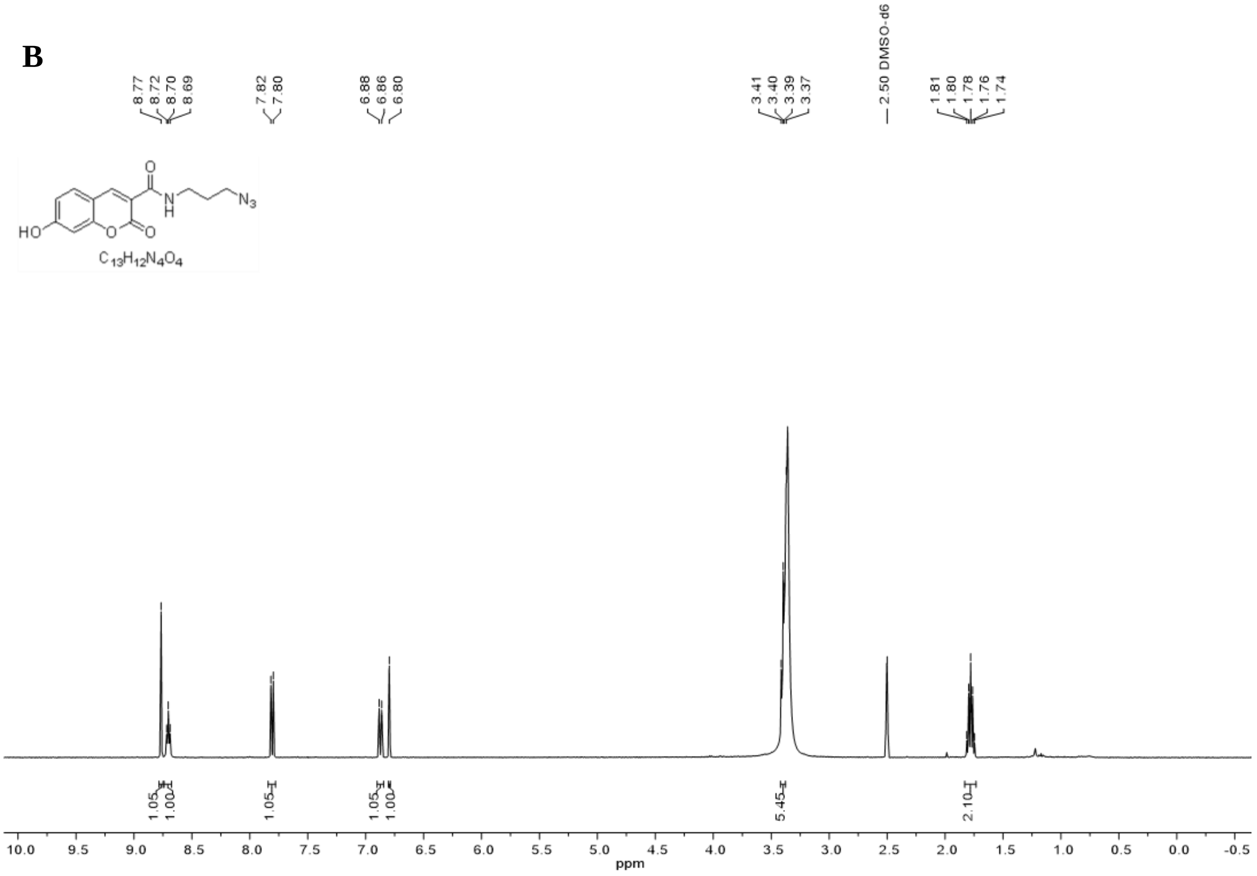

A - MS (ESI) *m/z*: cal. for $C_{13}H_{13}N_4O_4$ [M+H]+ 289,09 found 289,04. MS (ESI) *m/z*: cal. for $C_{13}H_{12}N_4NaO_4$ [M+Na] 311,08 found 310,91. **B -** ^{1}H NMR (400 MHz, DMSO-*d6*) of azide (7).

Supplementary Information 3: NMR and MS data for compound (9)

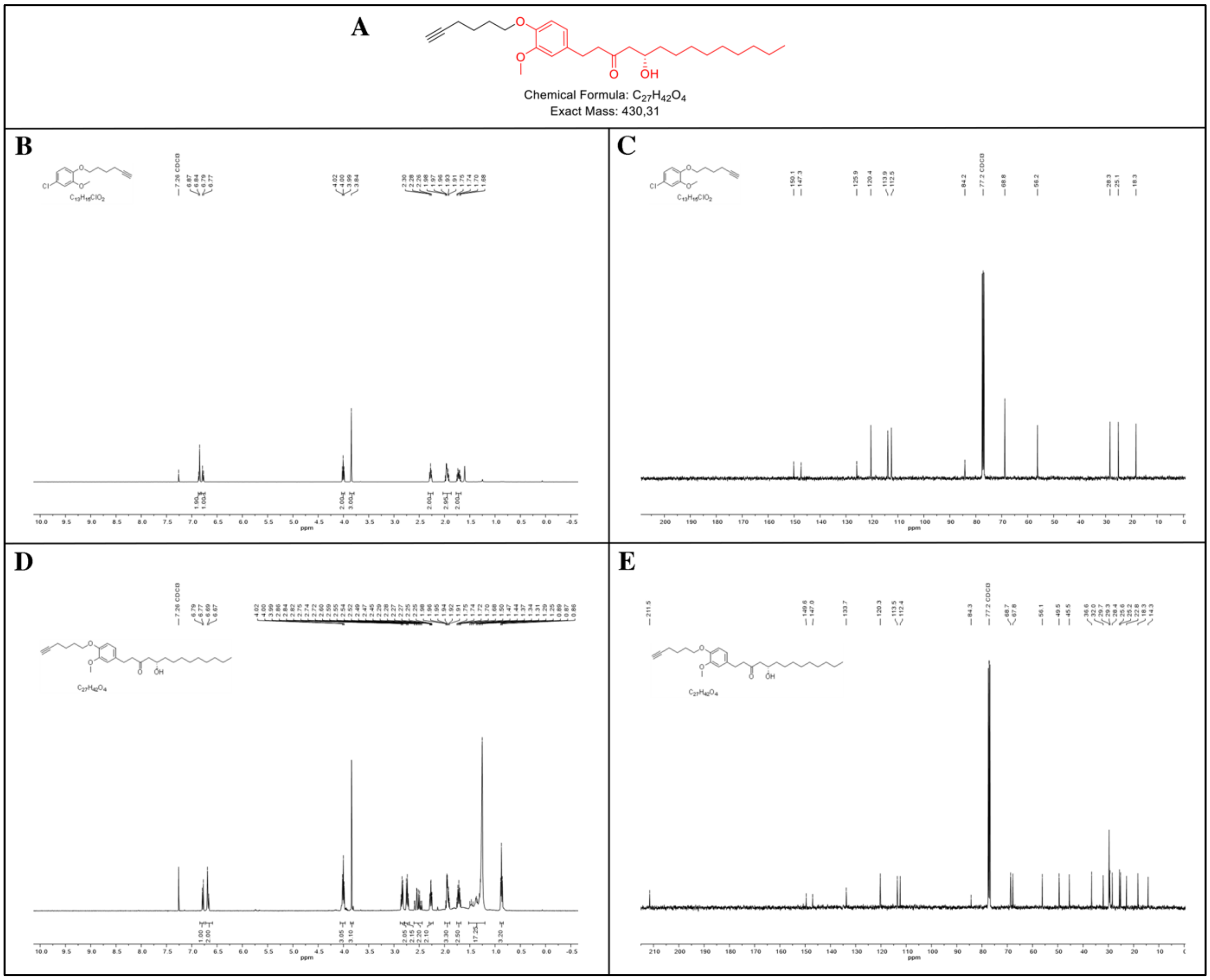

A - MS (ESI*)* *m/z:* cal. for $C_{27}H_{42}NaO_4$ [M+Na] 453,30 found 453,25. MS (ESI) *m/z*: cal. for $C_{27}H_{42}KO_4$ [M+K] 469,27 found 469,20. **B -** ^{1}H NMR (400 MHz, CDC_{l3}) of compound **9a**. **C -** ^{13}C NMR (101 MHz, CDC_{l3}) of compound **9a**. **D -** ^{1}H NMR (400 MHz, CDC_{l3}) of compound **9b**. **E -** ^{13}C NMR (101 MHz, CDC_{l3}) of compound **9b**.

Supplementary Information 4: NMR and MS data for LSPN280

A

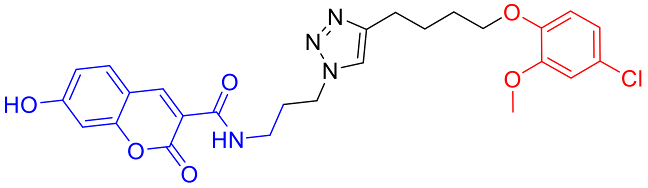

Chemical Formula: $C_{26}H_{27}ClN_4O_6$
Exact Mass: 526,16

B

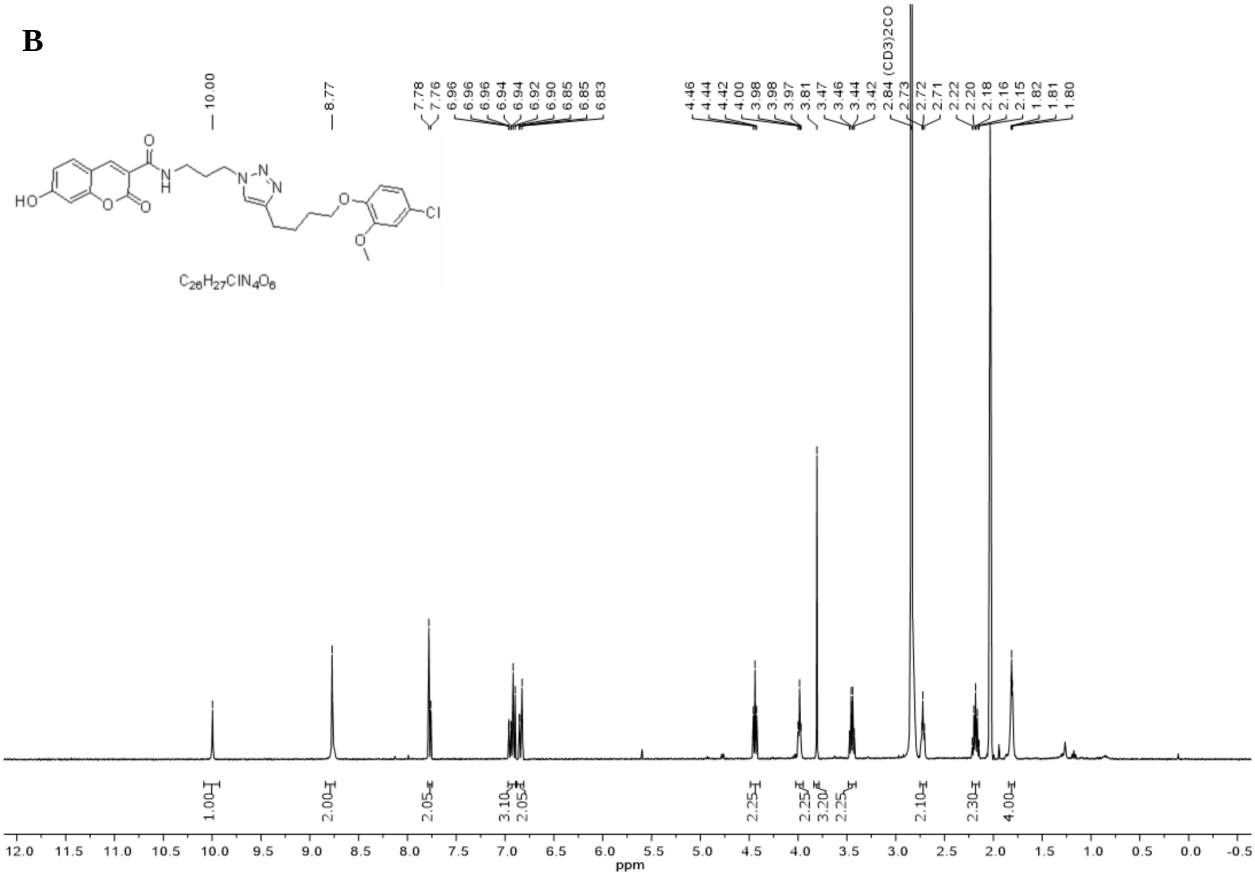

A - MS (ESI) *m/z*: cal. for $C_{26}H_{28}ClN_4O_6$ $[M+H]^+$ 527,17 found 527,17.**B -** ^{1}H NMR (400 MHz, Acetone-*d6*) of LSPN280.

Supplementary Information 5: NMR and MS data for LSPN281

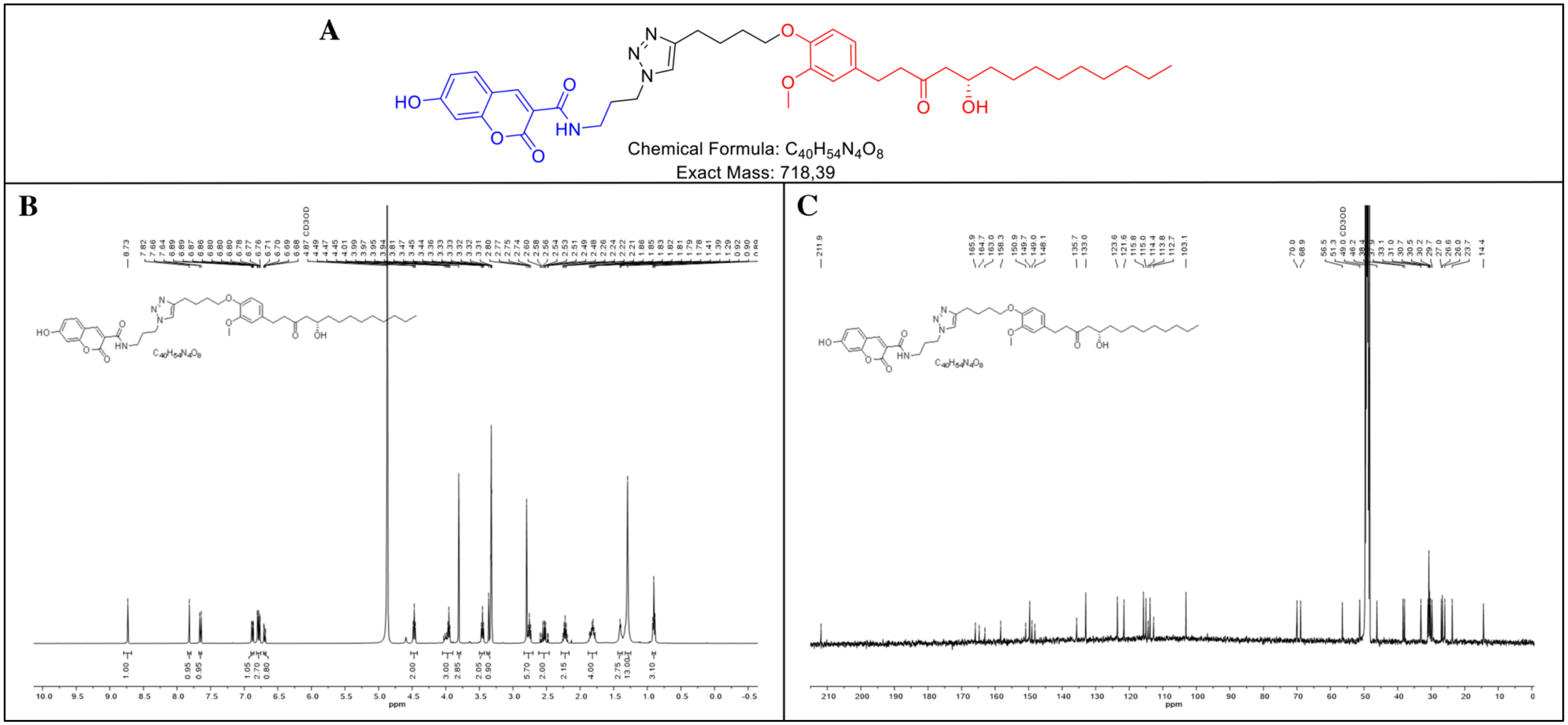

A - MS (ESI) *m/z*: cal. for $C_{40}H_{53}N_4O_8$ [M-H]- 717,4 found 717,3. **B -** ^{1}H NMR (400 MHz, CD_3OD) of LSPN281.

Supplementary Information 6 - Composition of images from epifluorescence HTS.

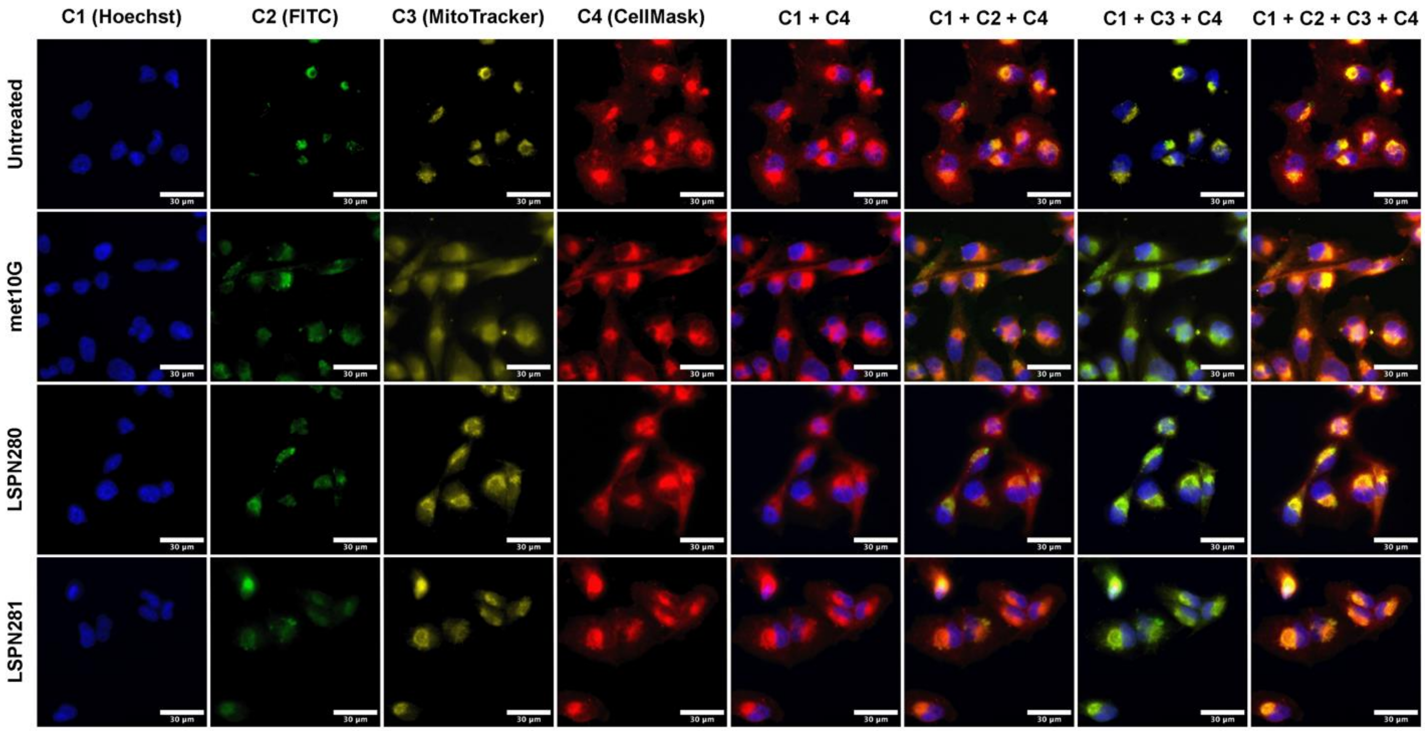

MDA-MB-231 cells were stained with Hoechst (nuclei, blue), MitoTracker (mitochondria, yellow) and CellMask (membrane, red) and treated with [10]-gingerol coumarin triazole hybrids (green). Images acquired in a high-content screening epifluorescence microscope (ImageXpress Micro XLS, Molecular Devices). Composites show the overlaid channels as described. Scale bar: 30 µm.

Supplementary Information 7 - Composition of confocal imaging and colocalization with mitochondria.

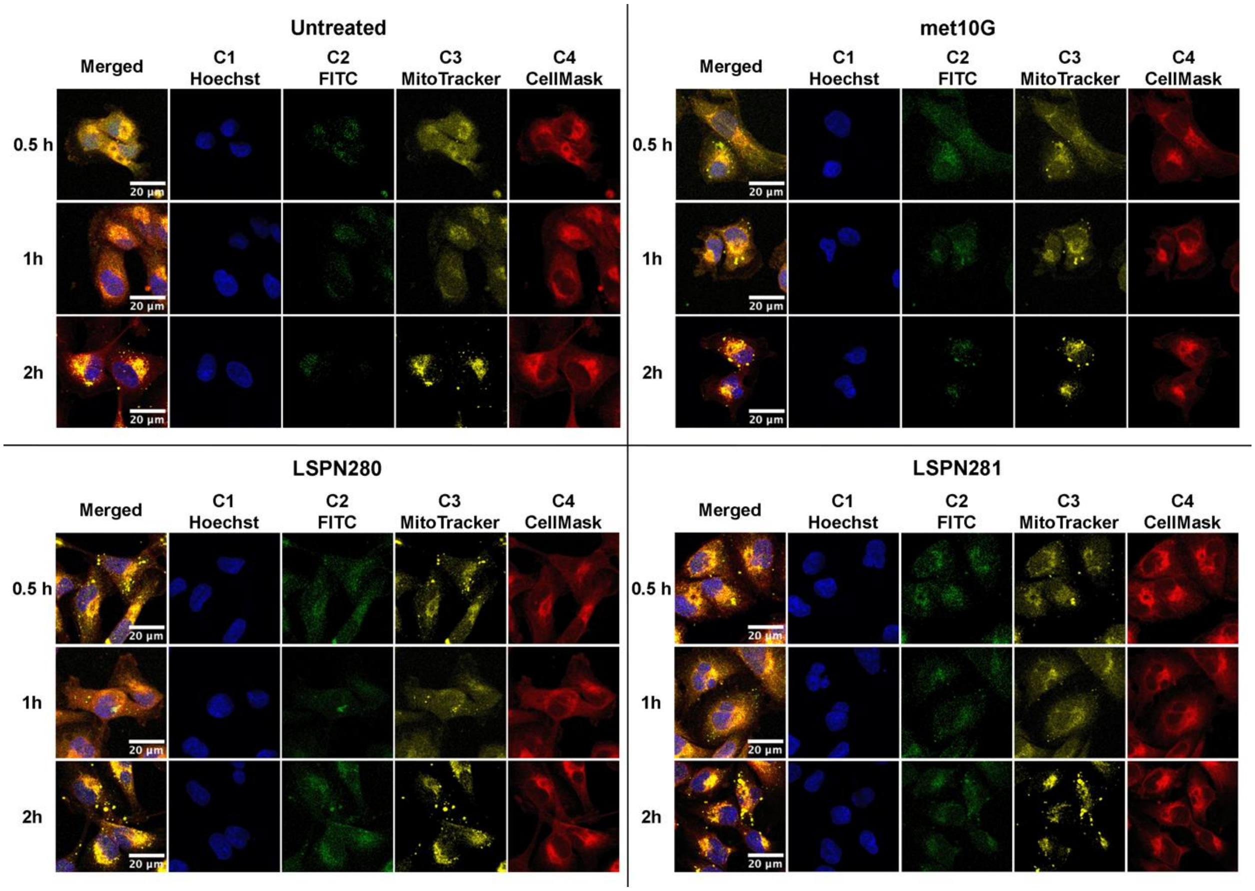

MDA-MB-231 cells were stained with Hoechst (nuclei, blue), MitoTracker (mitochondria, yellow), and CellMask (membrane, red) and treated with [10]-gingerol coumarin triazole hybrids (green). Each hybrid was tested at three-time points (0.5, 1, and 2 hours), and images were acquired in a confocal microscope (Zeiss LSM 880 AirySCAN, Carl Zeiss). 2D panels were assembled with merged images containing the four overlaid channels. Scale bar: 20 µm.
